# Screening and identification of host cellular factors interaction with immediate-early protein ICP22 of herpes simplex virus type 1

**Published:** 2011-01-10

**Authors:** Junji Xing, Fusen Lin, Meili Li, Shuai Wang, Hong Guo, Chunfu Zheng

**Affiliations:** 1 State Key Laboratory of Virology, Wuhan Institute of Virology, Chinese Academy of Sciences, Wuhan 430071, PR China

## 

Herpes simplex virus type 1 (HSV-1) is a common and widely studied human pathogen that can replicate in epithelial cells and other cells of the host or alternatively can remain latent in peripheral neurons. ICP22 consists of 420 residues and is encoded by a spliced mRNA transcribed from the US1 gene. It is necessary for efficient HSV-1 growth in animal models of infection as well as for efficient *in vitro* growth in some, but not all, cultured cells. For example, ICP22 mutants grow well in African green monkey kidney (Vero) cells, but not in human embryonic lung (HEL) cells. ICP22 is extensively phosphorylated during infection, primarily by UL13 and another viral protein kinase, US3. In addition to inducing the modification of the host cell RNA Pol II, several other functions have been attributed to ICP22; these functions include the induction of certain viral L genes, the alteration of cell cycle-related proteins, and the determination of virion composition. It is clear that ICP22 is a multifunctional protein localized to the nucleus of infected cells, however, the host cellular factors of ICP22 as well as the biological functions of their interactions are still little known. In the present study, an yeast two-hybrid system was applied to identify the host cellular factors of ICP22 and five target candidates were yielded: (1) TATA box binding protein -associated factor (TAF1); (2) TAO kinase 3 (TAOK3); (3) Alpha thalassemia/mental retardation syndrome X-linked (ATRX); (4) Cyclin-dependent kinase 9 (CDK9); (5) Ras association domain family member 9 (RASSF9); (6) occludin/ELL domain containing 1 (OCEL1). To confirm some of the interactions by co-localization in living cells, ICP22 and two candidate targets were tagged with enhanced cyan fluorescent protein (ECFP), enhanced yellow fluorescent protein (EYFP), respectively. Upon cotransfection of COS-7 cells, RASSF9-EYFP and OCEL1-EYFP both co-localized with ICP22-ECFP in distinct nuclear domains, indicating they are host cellular factors interaction with viral ICP22 under physiological conditions.

